# The effects of fake reviews during stepwise topic movement on shopping attitude in social network marketing

**DOI:** 10.1016/j.mex.2023.102461

**Published:** 2023-10-30

**Authors:** Masoumeh Hosseinzadeh Shahri, Farideh Haghbin, Yousef Qaseminezhad Raeini, Narjes Monfared

**Affiliations:** a Department of Management, Alzahra University, Tehran, Iran; b Department of Linguistics, Alzahra University, Tehran, Iran; c Department of Mathematics, Shahid Bahonar University of Kerman, Kerman, Iran

**Keywords:** Discourse analysis, Social network marketing, Integrated method, Integrated method

## Abstract

Although the influence of consumer reviews is increasing in weight, far-flung consumer comments on social networks are a retrogressive problem, disturbing users' attention in reviews studies by interpreting them as misleading messages. The need for investigating the unknown meaning layers of fake reviews in the stepwise topic movement of conversations and examining the effects of the fake reviews on consumers’ shopping attitudes encouraged us to adopt an integrated approach to marketing and discourse analyses.

• Qualitative analysis: To qualitatively investigate the stepwise topic movement of fake reviews in each sampled conversation of the research, three phases were taken into consideration: firstly, *identification of topic opening*; then, *topic closing procedure*; and finally, *the topic switch toward topic drift*.
• Quantitative investigation: we develop a questionnaire using multidisciplinary research variables. Then, the reliability and validity of the questionnaire were assessed using Cronbach's alpha and convergent and discriminant values, respectively. After that, the questionnaire was evaluated among a research sample. The data was analysed based on structural equation modeling (SEM) and machine learning (ML).
• Conclusion: It was found that fake reviews using topic coherence and grammatical-lexical cohesion mechanisms had positive effects on shopping attitudes. Moreover, fake reviews using topic drift mechanisms influenced consumers’ shopping attitudes.

## Graphical abstract

**Figure:**
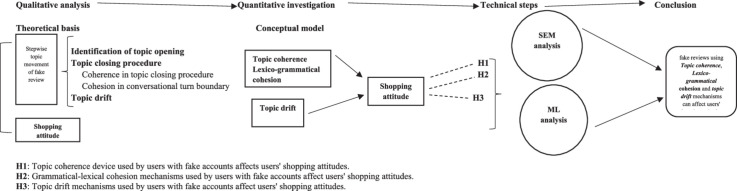

Specifications Table

**Table:** 

Subject area:	Economics and Finance
More specific subject area:	*Marketing, and Discourse analysis*
Name of your method:	*Integrated method*
Name and reference of original method:	*Qualitative investigation (discourse analysis); Quantitative analysis (SEM, ML)*
Resource availability:	*NA*

## Introduction

Consumer-based information in the form of online reviews is valuable for potential customers and, therefore, represents an essential element in the shopping attitude [1]. In recent years, there has been an increased interest in developing computational tools that efficiently detect disinformation using machine learning and deep learning techniques [[2], [3], [4]]. Among different approaches to fake news detection in Content Analysis models [5,6] and also marketing approaches in the purchase decision process [[7], [8], [9], [10]], language-based approaches have turned out to be promising. 'Language-based' in a broad sense includes a variety of approaches, such as those that employ traditional linguistic features, readability features, style-based features, discourse, and rhetorical features, or those that draw on word embedding techniques. Despite their success, however, their detection is based on latent features that are not humanly interpretable and cannot explain why a review was detected as a fake review. Generally speaking, linguistics research is concerned with recognizing trends and patterns in the discourse as narratives underlying this social interaction. Although the achievements of these studies are remarkable, the discourse of fake review – such as coherency and the topic drift of fake review in stepwise movement- and its effects on shopping attitudes have not been dealt with in depth in social network marketing.

Coherency as a dynamic process arises from mutual efforts of the communicator and addressee on a topic [[11], [12], [13]]. This phenomenon is more observable in online discussions where participants are not face-to-face [14]. As a discourse-as-product, coherence is a linguistic phenomenon realized on the surface of discourse by various linguistic devices used to connect different parts of a discourse. Although each conversation starts with a specific topic, and the participants are involved in the conversation [15], the topic ending the conversation is usually far from the initiating topic. In stepwise movement, “any next utterance is built in such a way as to be on-topic with a last” ([16], p. 300). Also, topics follow one another in a three-phase structure of opening, development, and closing or drifting; a topic gives way to the next, and so on. Accordingly, we applied an integrated model [17] to investigate which factors of topic coherence and lexical-grammatical cohesion are used by users with fake accounts that affect users' shopping attitudes in online written conversations and also which topic drift elements are applied by users with fake accounts in online reader-reader interactions that affect users’ purchasing attitude.

Addressing fake reviews is crucial for maintaining the credibility of online review systems and ensuring consumers can make informed choices based on reliable information. In this context, discourse-based methodologies, guided by a discursive lens, offer novel avenues for grasping marketing as both an academic discipline and how marketing scholars perceive and investigate topics within this domain of study. Accordingly, our research is primarily focused on examining how fake reviews within online conversations impact consumers' shopping attitudes. We place a particular emphasis on delving into the previously unexplored layers of meaning embedded within these fake reviews and their contribution to the progression of topics in online discussions. Therefore, we are aimed to investigate which factors of topic coherence and lexical-grammatical cohesion are used by users with fake accounts that affect users' shopping attitudes in online written conversations and also which topic drift elements are applied by users with fake accounts in online reader-reader interactions that affect users’ purchasing attitude. Our study addresses a notable knowledge gap related to the influence of fake reviews, leveraging mechanisms like topic coherence, grammatical-lexical cohesion, and topic drift, on consumers' shopping attitudes. In summary, our investigation aims to reveal concealed aspects of fake reviews and their consequential impact on shopping attitudes, thereby enriching our understanding of their role in online discourse and consumer decision-making processes. In this regard, our research employs an integrated approach, incorporating both qualitative and quantitative methods, along with structural equation modeling (SEM) and machine learning (ML). This integration is relevant because it allows us to comprehensively explore the multifaceted aspects of our research topic, enhancing the depth and robustness of our analysis.

## Methodology

This study was conducted from October 2021 to April 2022, when Instagram marketing in Iran grew following the outbreak of the coronavirus. Instagram was less filtered during than other social networks the post-corona period; hence, it was more widely used among groups and organizations in the Islamic Republic of Iran.

The methodological approach taken in this study is a mixed methodology. For quantitative analysis, a language-based model derived from the multidisciplinary integrated approach to marketing and discourse analysis was obtained by investigating the purposed sample in order to extract different sub-classes. Accordingly, data sampling has been purposely performed based on the publicly published reviews of Digikala as the most famous Holding company in Iran–up to 3000 reviews- on Instagram.

In this regard, we employed several knowledge-based techniques allowing us to identify fake accounts using criteria ranging from following/like ratios, followers' quality, Instagram account owner reliability, and others. Then, for quantitative investigation, a questionnaire was developed according to multidisciplinary research variables. The reliability and validity of the questionnaire were assessed using Cronbach's alpha and convergent and discriminant values, respectively.

After measuring the validity and reliability of the questionnaire, it was distributed among 369 individuals. Finally, the data were analysed both SEM and ML.

### Qualitative investigation

To qualitatively investigate the stepwise topic movement of fake reviews in each sampled conversation of the research, three phases were taken into consideration: firstly, *identification of topic opening*; then, *topic closing procedure (coherence and cohesion in topic movement)*; and finally, *the topic switch toward topic drift* [15]. *Topic opening* occurred when the brand shared designated content, prompting users to contribute their perspectives. As the conversation advances, *topic movement* occur when new discussions emerge among participants, yet it maintains continuity with the recently introduced subject. Furthermore, there are instances of boundary topical movement, where the conclusion of one topic gives way to the initiation of another, even including responses related to unrelated reviews. *Topic drift* takes place when the central topic is temporarily neglected or even permanently abandoned. The discourse variables of coherence, including cohesion and topic drift, were investigated following the further clarification of the multidisciplinary research variable of *consumer attitude.*

#### Identification of topic opening

While Halliday [18] emphasizes the significant role of topics in connecting utterances to their textual context, Daneš [19] points out that the level of discourse coherence is influenced by the interconnectivity of themes across different sentences. Razeghi et al. [14] argue that coherence in social network conversations encompasses subcategories related to relevance in visual text, verbal text, and multimodal discourse.

#### Topic closing procedure

##### Coherence in topic closing procedure

A stepwise movement is the development of a topic, whereas boundary movement concerns the limits between topics. Sacks [16] distinguishes between (a) stepwise topical movement, in which one topic flows into another such as all relevant answers of reviews in various forms as and (b) boundary topical movement in which the closure of one topic is followed by the initiation of another ([20], p. 165) including related response reviews to unrelated ones.

##### Cohesion in conversational turn boundary

**Fig. 1:**
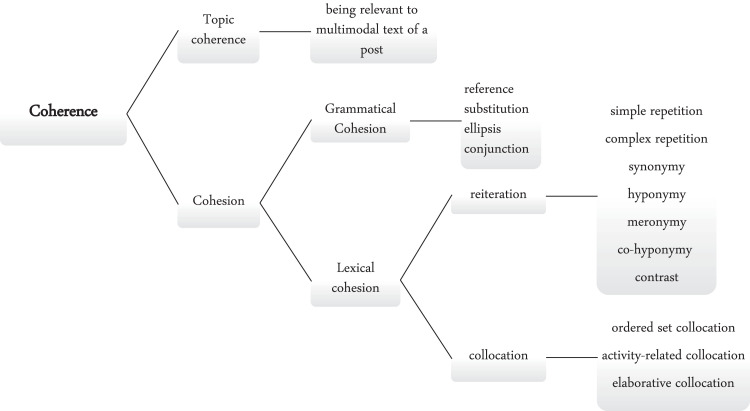
Coherence mechanisms of fake reviews.

**Fig. 2:**
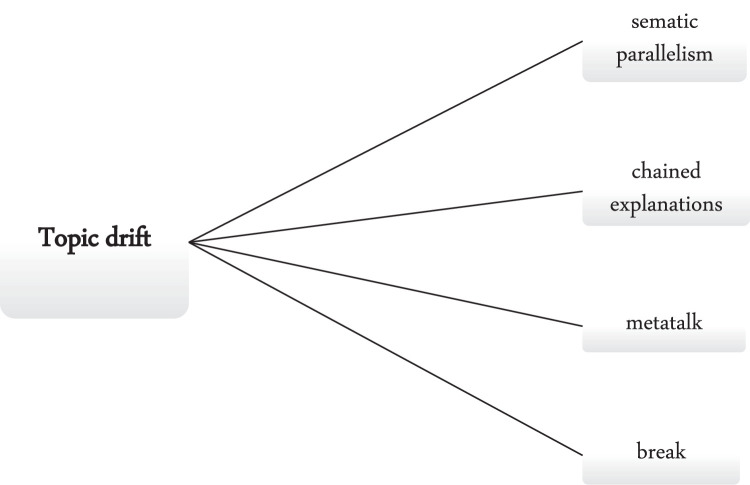
Topic drift mechanisms of fake reviews.

**Fig. 3:**
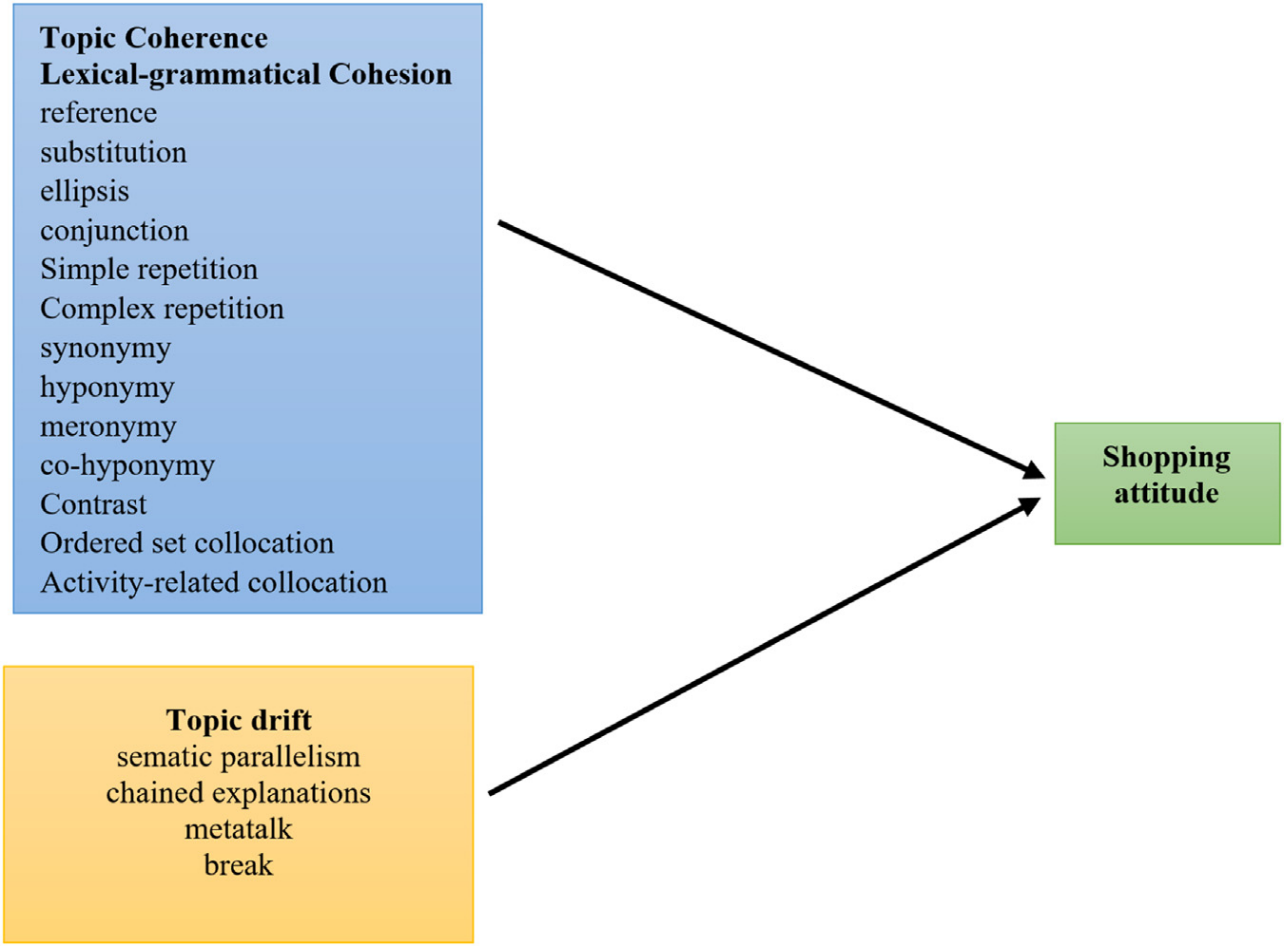
Conceptual model of research.

**Fig. 4:**
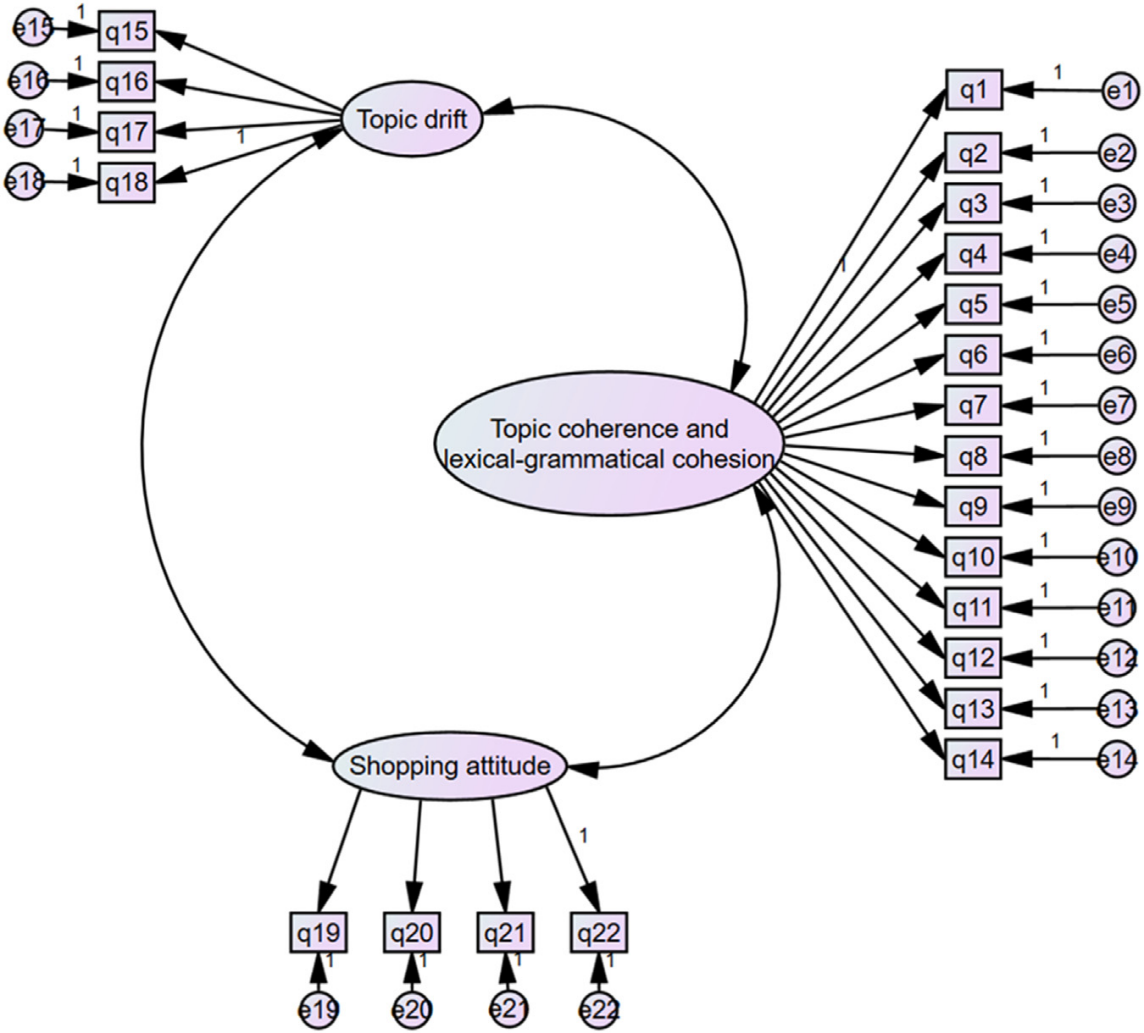
Results of the direct hypotheses of structural model.

Cohesion has been proposed to be the structural relations on the text surface, while coherence is the structural relations underlying the surface [[21], [22], [23]]. There are two ways by which cohesive ties are created: lexical and grammatical cohesion [24]. Grammatical cohesion involves grammatical cohesive ties categorized into four main groups: reference, substitution, ellipsis, and conjunction. Moreover, Halliday and Hasan [24] consider both reiteration and collocation in the analysis of cohesion. Reiteration includes seven subclasses: 1. Simple repetition occurs when an item is repeated either in an identical form or with no other than a simple grammatical change. 2. In complex repetition, the items may be identical but serve different grammatical functions, or they may not be identical but share a lexical morpheme. 3. Following McCarthy [25], the term equivalence is called synonymy. 4. Generalization has been referred to as a superordinate or hyponymic relation. 5. Specification refers to the relation between an item and a more specific item as meronymy. 6. Co-specification includes the relation between two items having a common general item as co-hyponymy or co-meronymy. 7. Contrast refers to the relation between an item and another item having an opposite meaning as antonymy, opposition, or complex repetition or paraphrase. Similarly, Collocation involves three subclasses: 1. Ordered set includes members of ordered sets of lexical items, for example, colors, numbers, months, days of the week, and the like. 2. In Activity-related collocation, we cannot construct watertight rules to say which items are related and which are not, but some previous studies on native speakers' preferences may help us understand and classify these complex relations. 3. Elaborative collocation is an association between items that cannot be classified as an ordered set or activity-related collocation, and it is only created during the conversation [26]. The diagram below shows the coherence mechanisms of fake reviews (Fig. 1, Fig. 2, Fig. 3, Fig. 4).

#### Topic drift

Topic drift occurs when the global topic is temporarily neglected or even permanently abandoned. Discourse then develops according to what is associatively closest or “easiest to say next” (Agar & Hobbs, 1986, p. 231) rather than to what the speaker's projected goals of the global topic demand. In topic drift, each segment coheres with the preceding and the following segment, but where the conversation ends up is quite far from where it started. Topic drift occurs by means of successive small modifications in the topic [27].

According to Hobbs [28], the subsequent discourse segment is either on-topic or shifts the topic through: (a) semantic parallelism, which includes the introduction of different entities with the same properties as those already mentioned, or other properties of the same entities. (b) chained explanations or explanations refer to the topics in their own right. Explanations expand on the topic at hand by explaining a previous proposition. (c) Metatalk (evaluation) is used as a way of introducing a new topic. (d) Break generally changes the topic ([29], p. 4). The topic drift mechanisms of fake reviews are summarised in the figure below.

### Consumer attitude

In social networks, when consumers are more involved with the product, they should be more worried about the consumer attitude toward shopping [30] and have a higher risk aversion ([31], p. 45). Meanwhile, reliable information has a great significance in guiding consumers' purchase decisions [32,33]. Consumers' perceptions about an advertised brand are (negatively) affected by the presence of fake reviews, regardless of the source's a priori credibility or individuals' a priori positive beliefs about the source. The path from source credibility to brand trust holds regardless of individuals' retaining a memory of the brand in an ad. This form of intentional blindness [34] is consistent with previous studies that documented people's unconscious processing of web advertising [35].

## Quantitative investigation

The current research employed a combination of structural equation modeling (SEM) and machine learning (ML) approaches. The initial phase of our results focused on utilizing SEM as a powerful tool for predicting the research model. Subsequently, we applied the ML approach to cluster various consumer segments. It's worth noting that both of these clustering algorithms fall under unsupervised machine learning methods. The integration of SEM and ML presents practical opportunities, enabling businesses to make well-informed decisions and enhance their strategies through data-driven consumer insights.

### Conceptual model

Based on the theoretical background just presented, the figure below shows the model proposed and submitted for the empirical test. The independent variables include Topic Coherence, Lexical-grammatical Cohesion, and Topic drift, and the dependent variable is the shopping attitude.

Presumably, the first main hypothesis was proposed as “topic coherence device used by users with fake accounts affects users' shopping attitudes.” Furthermore, our findings show our main second hypothesis stating that “grammatical-lexical cohesion mechanisms used by users with fake accounts affect users' shopping attitudes.” Regarding the subcomponents of grammatical-lexical cohesion, it can be stated that this hypothesis involves several secondary hypotheses suggesting that each of these devices, i.e., reference, substitution, conjunction, simple repetition, complex repetition, synonymy, hyponymy, meronymy, co-hyponymy and order set collocation, utilized by the users with fake accounts have a positive effect on users' shopping attitudes. However, other devices, including ellipsis, contrast, activity-related collocation, and elaborative collocation, used by users with fake accounts have adverse effects on users' shopping attitudes.

Finally, we believe that our research has probably usefully employed a third main hypothesis which states, “topic drift mechanisms used by users with fake accounts affect users' shopping attitudes.” As for the sub-categories of this hypothesis, it has to be noted that semantic parallelism, chained explanation, metatalk, and also break have had a negative effect on users' shopping attitudes.

### Questionnaire design

The research variables employed in this study have been subject to expert evaluation. The questionnaire comprises 22 questions, each corresponding to specific research variables, namely topic coherence, lexical-grammatical cohesion, topic drift, and shopping attitude. These variables are instrumental in investigating the nuances of consumer behavior within the digital realm, thereby yielding invaluable insights for the optimization of marketing strategies and the enhancement of the holistic consumer experience. In formulating the research inquiries, we utilized a 5-option Likert scale, a widely recognized measurement tool. This scale presents respondents with a series of statements and response options, enabling them to express their level of agreement or disagreement with specific subjects or concepts, whether positively or negatively. In essence, this scale functions as a comprehensive assessment tool, streamlining the evaluation of respondents' attitudes and beliefs.

The questionnaire was analyzed in terms of their reliability, by means of the internal consistency (Cronbach's alpha) and also validity, considering convergent and discriminant validity. Reliability, initially assessed by Cronbach's alpha, was computed in two stages: considering the original items of each scale and after that, the exclusion of those with lower correlations with the scale. To uphold the content validity of our research instrument, we initiated the questionnaire development process by extensively reviewing pertinent literature. Subsequently, leveraging expert judgment, we meticulously refined the questionnaire by incorporating essential adjustments. During this critical phase, the questionnaire was meticulously reviewed and scrutinized by a panel of six experts, who provided invaluable insights into the suitability of each item for effectively measuring the intended constructs.

### Statistical population

The statistical population of the present study consists of all people who are members of the social network Instagram. Respondents were instructed to consider reality while answering the questions with transparency and loyalty.

## Technical steps

The current research employed a combination of structural equation modeling (SEM), and machine learning (ML) approaches.

### Structural equation modeling analysis

SEM is analysed and interpreted in two phases, namely, the assessment of the measurement model where indicator items are examined and the assessment of the structural model, which involves examining the significance of the path coefficients, model of predictive relevance, and the like [36,33].

#### Measurement models

The tables below show the measurement model of coherence and cohesion and topic drift in shopping attitudes, which explains the factor loadings and reliability of the constructs based on the comparative fit index (CFI). CFI is an index of “good fit,” ranging from 0 to 1, which quantifies the proportional improvement in structural equation model fit over a “null” model (e.g., [[36], [37], [38], [39]]).

As can be seen, the study examined the mediating effect of topic coherence and lexical-grammatical cohesion, and topic drift on the influence of attitude on shopping and employed the internal consistency approach to determine the reliabilities of all the items of the constructs used in the study by using CFI. The covariance and correlations of the data indicate that all constructs differ from one another consequently; thus, discriminant validity is supported (See Table 1, Table 2, Table 3).

**Table 1:** Regression Weights: (Group number 1 - Default model).

			Estimate	S.E.	C.R.	P	Label
V2	<—	Coherence & Cohesion	1.000				
V3	<—	Coherence & Cohesion	.890	.079	11.273	***	
V4	<—	Coherence & Cohesion	.845	.076	11.157	***	
V5	<—	Coherence & Cohesion	1.275	.089	14.302	***	
V6	<—	Coherence & Cohesion	1.228	.087	14.071	***	
V7	<—	Coherence & Cohesion	1.217	.087	13.954	***	
V8	<—	Coherence & Cohesion	.960	.087	10.980	***	
V9	<—	Coherence & Cohesion	.965	.085	11.382	***	
V10	<—	Coherence & Cohesion	.788	.080	9.906	***	
V11	<—	Coherence & Cohesion	1.014	.090	11.288	***	
V12	<—	Coherence & Cohesion	1.127	.091	12.448	***	
V13	<—	Coherence & Cohesion	.956	.087	10.971	***	
V14	<—	Coherence & Cohesion	.948	.083	11.427	***	
V18	<—	Topic drift	1.000				
V17	<—	Topic drift	.990	.022	45.383	***	
V16	<—	Topic drift	.710	.038	18.497	***	
V15	<—	Topic drift	.700	.038	18.216	***	
V22	<—	Shopping Attitude	1.000				
V21	<—	Shopping Attitude	2.100	.338	6.219	***	
V20	<—	Shopping Attitude	.468	.142	3.308	***	
V19	<—	Shopping Attitude	1.334	.225	5.917	***	
V1	<—	Coherence & Cohesion	.666	.097	6.838	***

**Table 2:** Standardized regression weights: (Group number 1 - Default model).

			Estimate
V2	<—	Coherence & Cohesion	.655
V3	<—	Coherence & Cohesion	.649
V4	<—	Coherence & Cohesion	.641
V5	<—	Coherence & Cohesion	.863
V6	<—	Coherence & Cohesion	.845
V7	<—	Coherence & Cohesion	.837
V8	<—	Coherence & Cohesion	.630
V9	<—	Coherence & Cohesion	.656
V10	<—	Coherence & Cohesion	.561
V11	<—	Coherence & Cohesion	.650
V12	<—	Coherence & Cohesion	.728
V13	<—	Coherence & Cohesion	.629
V14	<—	Coherence & Cohesion	.659
V18	<—	Topic drift	.961
V17	<—	Topic drift	.976
V16	<—	Topic drift	.716
V15	<—	Topic drift	.710
V22	<—	Shopping Attitude	.392
V21	<—	Shopping Attitude	.843
V20	<—	Shopping Attitude	.215
V19	<—	Shopping Attitude	.537
V1	<—	Coherence & Cohesion	.377

**Table 3:** Covariances: (Group number 1 - Default model).

			Estimate	S.E.	C.R.	P	Label
Coherence & Cohesion	<–>	Topic Drift	.449	.052	8.619	***	
Coherence & Cohesion	<–>	Shopping Attitude	.184	.036	5.076	***	
Topic Drift	<–>	Shopping Attitude	.271	.053	5.155	***	

The table above shows that the relationship between the latent variables is significant.

Furthermore, the table below displays the standardized correlation coefficient of the relationships between the variables. In general, the standardized correlation coefficient of the relationships between the variables, which is often 0.5, indicates the high intensity of this relationship.

Table 4 illustrates the findings of the positive and direct hypotheses of this study. All of the hypotheses were accepted.

**Table 4:** Correlations: (Group number 1 - Default model).

			Estimate
Coherence & Cohesion	<–>	Topic Drift	.660
Coherence & Cohesion	<–>	Shopping Attitude	.591
Topic Drift	<–>	Shopping Attitude	.528

#### Structural model

A structural model represented, estimated, and tested a theoretical network or (mostly) linear relation between variables [33]. Also, we tested hypothesized patterns of directional and nondirectional relations among a set of observed (measured) and unobserved (latent) variables.

The structural model above illustrates the internal relationship between research variables. The indices of the latent variable “Topic Coherence and Lexical Grammatical Cohesion” were evaluated with 14 independent variables. Among these indicators, Topic Drift with one independent variable, Lexical Cohesion with seven independent variables, and Grammatical Cohesion with six variables were measured. Also, the indicators of Topic Drift as a latent variable were measured by four independent variables. Finally, the latent variable of Shopping attitude was examined by four independent variables.

In general, it can be observed that the relationship between research variables is positive and direct. Furthermore, there is a positive and direct correlation between the latent variable of “Topic Coherence and Lexical-grammatical Cohesion” with “Shopping Attitude,” “Topic Drift” with “Topic Coherence and Lexical-grammatical Cohesion,” and also “Topic Drift” with “Shopping Attitude.” It has to be noted that the correlation between the two latent variables of “Topic Coherence and Lexical-grammatical Cohesion” and “Shopping Attitude” is more intense.

### Machine learning analytics

This study also aimed to examine the relationship between 369 Instagram users and their feedback on this social network using 26 questions, four of which addressed the users' personal information, including *age, level of education, the extent of users' activity on Instagram*, and *gender*. No preprocessing steps were required as there was no missing value in the data set.

**Fig. 5:**
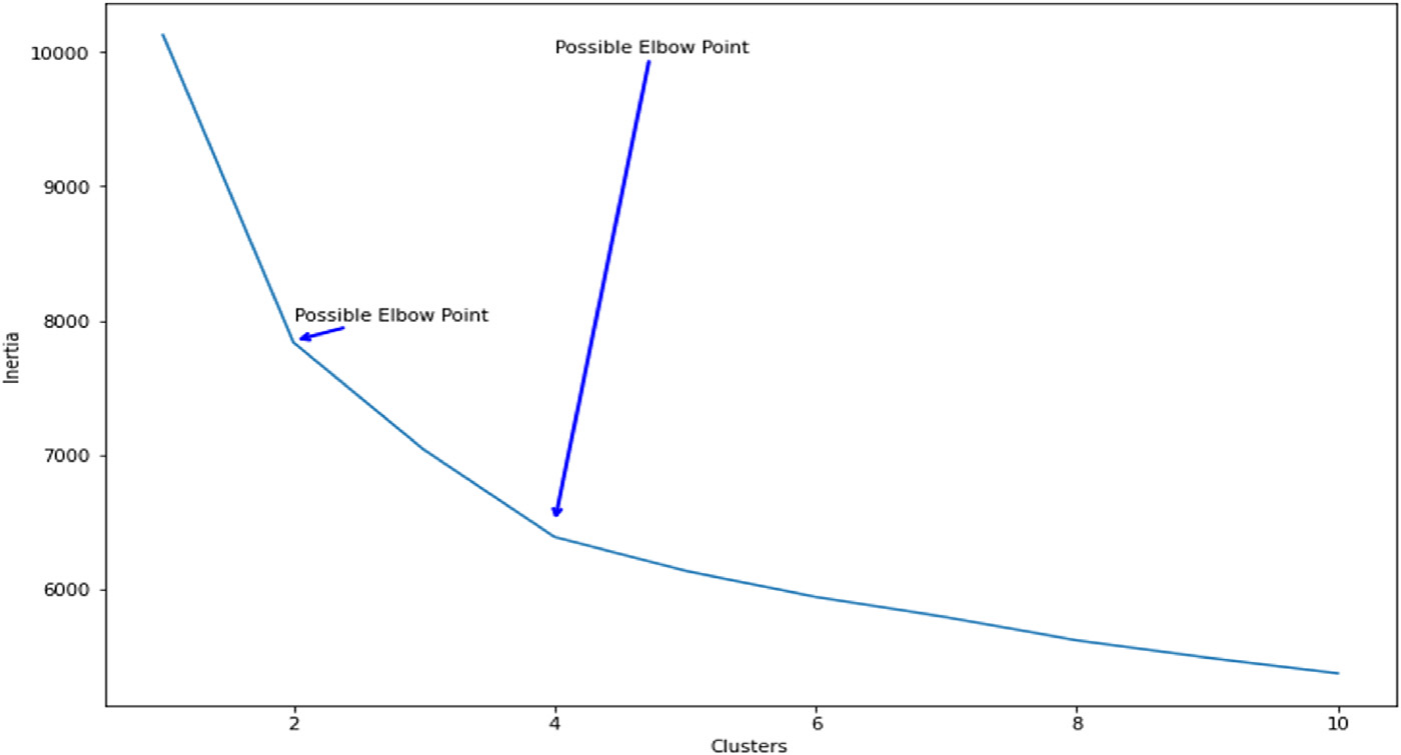
Searching elbow.

Accordingly, unsupervised learning uses artificial intelligence (AI) algorithms to identify patterns in datasets containing data points that are neither classified nor labeled. In this regard, clustering as a data mining technique groups unlabeled data by their similarities or differences. Meanwhile, exclusive clustering is a form of grouping stipulating a data point existing only in one cluster [30]. After examining and tuning different unsupervised algorithms, the K-means clustering model was selected as the most efficient and effective technique. In this regard, a fundamental step for any unsupervised algorithm is determining the optimal number of clusters for the collected data. The elbow method was used in this study to detect the most appropriate and optimal number of clusters for the concerned dataset, according to which 2 and 4 clusters were suggested (Fig. 5). To choose one of these numbers as the final number, some metrics of unsupervised models were adopted.

To measure the distinctness of clusters, the K-means clustering model was used to divide the participants into two groups with labels 0 and 1, containing 316 and 53 members, respectively.

**Fig. 6:**
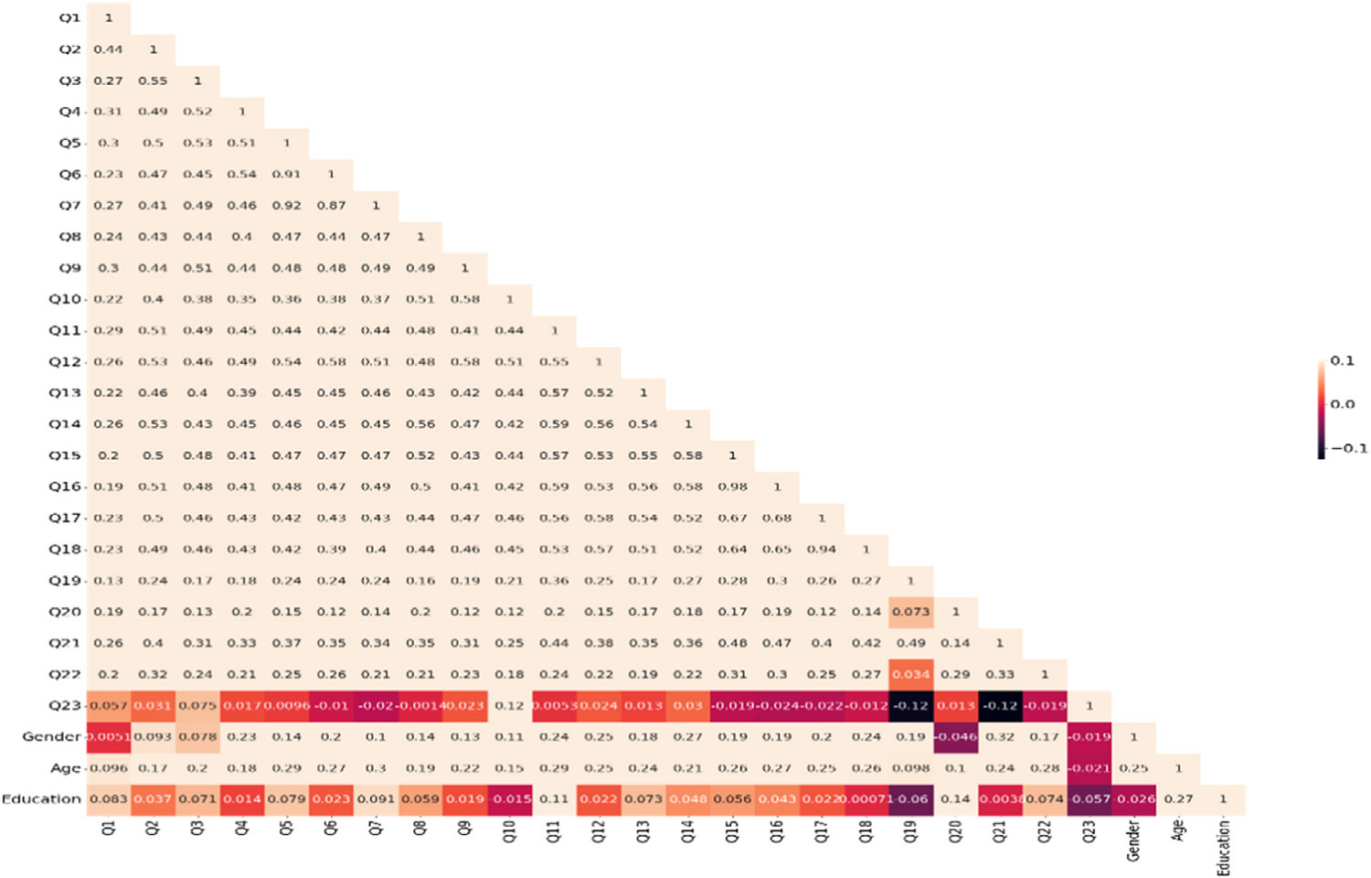
Heatmap of correlation among concerned variables.

Moreover, the correlation among the concerned variables is calculated and demonstrated in the following Heatmap (Fig. 6).

This figure shows the inverse relationships between *the extent of users' activity on Instagram* and the following variables *coherence, cohesion* (including *complex repetition, synonymy*, and *collocation*), *topic drift* (including *semantic parallelism, chained explanations, metatalk,* and *break*), *user's attitude*, and *gender*. However, the intensity of the relationships is limited by the displayed values. Furthermore, there is an inverse relationship between *gender* and *user's attitude* and between *age* and *user's attitude*. Although the relationships between *user's attitude* and *coherence* and between *attitude* and *gender* are significant, their relationships are not intensive. Finally, the relationship between the rest of the variables is obtained directly and significantly.

## Conclusion

Fake reviews are deliberately fabricated expressions that have the potential to lead to financial repercussions and a decline in customer loyalty. These reviews are not always straightforwardly binary, as some may contain elements of truth alongside falsehoods. Previous research has primarily focused on binary classification and has examined the impact of fake reviews using content analysis models and marketing approaches. However, these investigations have yet to fully leverage an integrated model that encompasses various discourse-based approaches, such as analyzing topic coherence, cohesion, and topic drift, to comprehensively understand their influence on consumer shopping attitudes.

Even though Fiedler and Kissling [8] points out the role of coherency in the detection of a fake review, extreme attention must be paid to the effects of topic coherency of fake reviews on shopping attitude. By examining the first main hypothesis, we reached the conclusion that fake reviews by using topic coherence devices can affect users' shopping attitudes. The most remarkable result to emerge from the data was that users with fake accounts positively affect other users by relatedly talking about a brand´s post. This finding reinforces the usefulness of topic coherence as a theory proposing that a coherent text must be consistent with the context in which it is created, and all parts of the text must be connected. Our findings appear to be well substantiated to confirm the second main hypothesis stating that fake reviews utilizing grammatical-lexical cohesion mechanisms can affect users' shopping attitudes. However, this hypothesis would lend itself well to involve several secondary hypotheses.

Finally, the most striking result from evaluating the third hypothesis is that fake reviews using topic drift mechanisms can affect users' shopping attitudes. Simply speaking, all of the topic drift mechanisms, including *semantic parallelism, chained explanations, metatalk*, and *break* were found to have a negative effect on users' shopping attitudes. Thus, to create a gap in the conversation, fake reviews mention what has already been mentioned in the reviews, repeat a sentence during the conversation, mention a new issue, or completely change the subject of the conversation.

In essence, these strategies are employed to manipulate online discussions, sway public opinion, or create the illusion of a more diverse and genuine conversation when, in reality, it may be orchestrated by a single entity with a specific agenda. It's important for readers to be aware of these tactics and critically assess online content, especially when encountering reviews or comments that appear suspicious or overly orchestrated.

This article was supported by the Iran National Science Foundation and Alzahra University, Tehran, Iran with a number of 99,016,793.

## Declaration of Competing Interest

The authors declare that they have no known competing financial interests or personal relationships that could have appeared to influence the work reported in this paper.

## Data Availability

The data that has been used is confidential.
